# Effective Swimmer’s Action during the Grab Start Technique

**DOI:** 10.1371/journal.pone.0123001

**Published:** 2015-05-15

**Authors:** Luis Mourão, Karla de Jesus, Hélio Roesler, Leandro J. Machado, Ricardo J. Fernandes, João Paulo Vilas-Boas, Mário A. P. Vaz

**Affiliations:** 1 Superior School of Industrial Studies and Management, Porto Polytechnic Institute, Vila do Conde, Portugal; 2 Centre of Research, Education, Innovation and Intervention in Sport, Faculty of Sport, University of Porto, Porto, Portugal; 3 Aquatic Biomechanics Research Laboratory, Health and Sports Science Centre University of the State of Santa Catarina, Florianópolis, Santa Catarina, Brazil; 4 Porto Biomechanics Laboratory, University of Porto, Porto, Portugal; 5 Institute of Mechanical Engineering and Industrial Management, Faculty of Engineering, University of Porto, Porto, Portugal

## Abstract

The external forces applied in swimming starts have been often studied, but using direct analysis and simple interpretation data processes. This study aimed to develop a tool for vertical and horizontal force assessment based on the swimmers’ propulsive and structural forces (passive forces due to dead weight) applied during the block phase. Four methodological pathways were followed: the experimented fall of a rigid body, the swimmers’ inertia effect, the development of a mathematical model to describe the outcome of the rigid body fall and its generalization to include the effects of the inertia, and the experimental swimmers’ starting protocol analysed with the inclusion of the developed mathematical tool. The first three methodological steps resulted in the description and computation of the passive force components. At the fourth step, six well-trained swimmers performed three 15 *m* maximal grab start trials and three-dimensional (3D) kinetic data were obtained using a six degrees of freedom force plate. The passive force contribution to the start performance obtained from the model was subtracted from the experimental force due to the swimmers resulting in the swimmers’ active forces. As expected, the swimmers’ vertical and horizontal active forces accounted for the maximum variability contribution of the experimental forces. It was found that the active force profile for the vertical and horizontal components resembled one another. These findings should be considered in clarifying the active swimmers’ force variability and the respective geometrical profile as indicators to redefine steering strategies.

## Introduction

It is known that the 15 *m* starting performance can differ amongst elite swimmers by only ~0.40 *s* [1,2], with a decisive effect on the final result in several competitive events. The grab and track starts used in ventral events are the most extensively studied techniques [3]: in the grab start, the swimmers’ hands grasp the front edge of the block (either between or at the outer edge of the feet) and in the track start swimmers position one foot on the front edge of the starting block and the other foot behind, with the possibility of placing the body weight toward the front edge or toward the rear of the block [2,4].

Some authors have studied the external forces that affect the swimmers’ movement on the starting block during the grab and/or track start techniques [4,5,6,7,8,9] by measuring the total anterior-posterior [4,5,6,7,8,9], vertical [4,8,9] and lateral reaction forces [9]. The vertical force applied into the block accelerates the swimmer’s centre of mass (CM) in the upward/downward direction, the anterior-posterior force generates propulsion mainly in the forward direction and the lateral force is essentially a controlling movement [10].

Despite the essential contribution of previous research regarding the external kinetics involved during ventral swimming starts, the process of interpreting and analysing data is still not as effective as it should be [8]. Based on fundamental mechanics, the forces applied on the starting block may be interpreted as being dependent upon the active forces and the body weight dynamical effects in each successive body position enabling to provide more accurate information about performance diagnosis [11]. Using Newton’s 3^rd^ law, the total ground reaction force exerted by the starting block on the swimmer $\left(\vec{GRF}\left(t\right)=\left(GRF_{h}\left(t\right),GRF_{v}\left(t\right)\right)\right)$, where it has been separated into its most relevant components accordingly to [10], is the opposite of the action force applied on the block surface, and it involves the swimmer’s muscular action and postural effect while moving (changing multi-segment configuration and CM position). Sometimes the vertical component of GRF is termed $\vec{N}$, the normal reaction, and the horizontal component is termed ${\vec{F}}_{s}$, the static friction. Returning to the swimming start block, the swimmer’s acceleration is defined through Newton’s 2^nd^ law as:

$$\vec{GRF}\left(\text{t}\right)+\vec{W}=\text{m}\cdot {\vec{a}}_{\text{swimmer}}\left(\text{t}\right)$$ (1)

Where $\vec{W}$, *m* and ${\vec{a}}_{swimmer}\left(t\right)$ are the swimmer’s weight, mass and ${\vec{\text{a}}}_{\text{swimmer}}\left(\text{t}\right)$ acceleration, respectively, being $\vec{GRF}$ applied at the halluces (feet) and $\vec{W}$ at the center of mass. In accordance, the total impulse or linear momentum increment $\left(\Delta \vec{p}\right)$, leading to CM kinematics classical description is defined as the time integral:

$$\int_{0}^{\tau }\left(\vec{GRF}\left(\text{t}\right)+\vec{W}\right)\cdot \text{dt}=\;\Delta \vec{\text{p}}$$ (2)

However, even in the absence of a swimmer’s active starting effort, impulse generation remains, which can be evidenced by considering the fall of a similar passive rigid body. Therefore, in this particular case, the $\vec{GRF}$ is simply a passive force, that is:

$$\vec{GRF}\left(t\right)={\vec{R}}_{Passive}\left(t\right)$$ (3)

The ${\vec{R}}_{Passive}\left(t\right)$ (the $\vec{GRF}\left(t\right)$ applied to the inertial structure of the swimmer’s body) should be considered in this formalism as the one generated by a falling inert rigid body. Keeping in mind these ideas, it is suggested to decompose $\vec{GRF}\left(t\right)$ in the general (and real) case into passive and active components, as:

$$\vec{GRF}\left(t\right)={\vec{R}}_{Passive}\left(t\right)+{\vec{R}}_{Active}\left(t\right)$$ (4)

where ${\vec{R}}_{Active}\left(t\right)$ is in the opposite direction to that of the propulsive force vector applied to the block by the swimmer’s muscular actions and ${\vec{R}}_{Passive}\left(t\right)$ is the same as in Eq 3.

The aim of this research is to decompose the $\vec{GRF}\left(t\right)$ into ${\vec{R}}_{Active}\left(t\right)$ and ${\vec{R}}_{Passive}\left(t\right)$ in the grab start, which is one of the most used ventral starting techniques [2,3,9,12]. This start technique is the most suitable to apply the force splitting formalism, since the swimmer’s body is in contact with the platform by means of the halluces-platform alignment whose centre should be the centre of pressure (COP). In fact, this particular geometry may be described as the CM rotation around the halluces lateral-medial axis, combined with the CM displacement along the anterior-posterior CM-COP direction. As this geometry is partly shared with the track start, a similar approach can be applied when the swimmer’s rear lower limb leaves the block. It is hypothesised that it is possible to decompose $\vec{GRF}\left(t\right)$ into its ${\vec{R}}_{Passive}\left(t\right)$ and ${\vec{R}}_{Active}\left(t\right)$ components, allowing researchers and coaches to better understand the real swimmer’s force generation contribution during the block phase.

## Material and Methods

### General description

Four working pathways were followed: (i) the experimented fall of a simple rigid body; (ii) the swimmers’ matrix of inertia determination; (iii) the development of a mathematical model to describe the outcomes of the rigid body fall experiment and its generalization to provide replacement of the calculated inertia; and (iv) the experimental start protocol and data analysis including the developed mathematical model. The first three steps were defined to achieve the transient swimmer’s angular positions during the starting movement in order to better understand the influence of the passive forces on start performance.

### Physical rigid body falling

**Fig 1:**
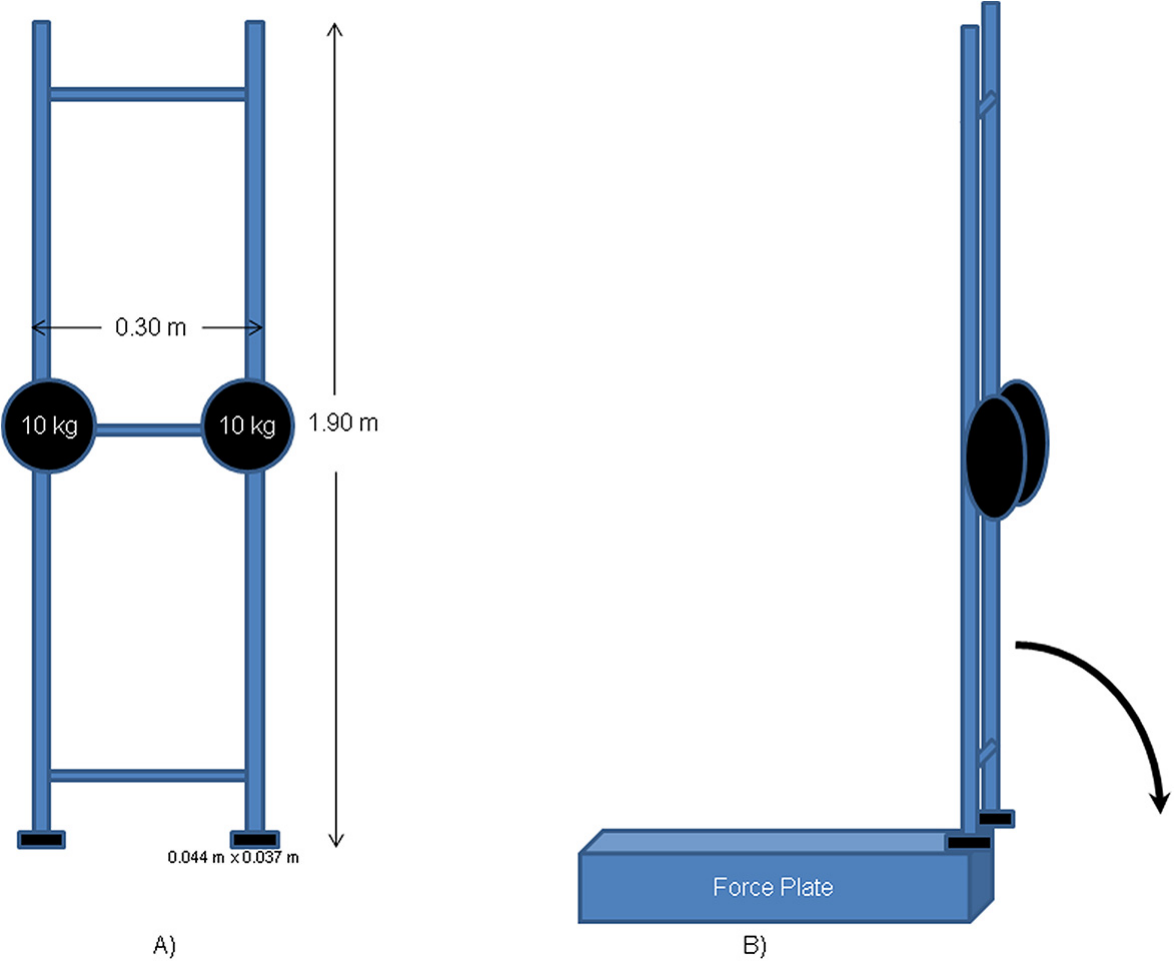
Scheme of a rigid body balanced at the force plate border: frontal view (A) and isometric falling rotation around the medial-lateral axis that contains the centre of pressure (B).

A rigid rectangular stainless steel structure (1.80 *m* in height, 0.30 *m* in width and 27 *kg* total mass) was used. The structure CM was located at 0.9 *m* height and two stainless steel masses (10 *kg* each) were fixed at that height in each structure side. The lower extremity structure’s was rectangular and divided into two contact surfaces (0.044 *m* x 0.037 *m*) (Fig 1A). To simulate the support of the swimmer’s feet, the structure was balanced at the front edge of a 3D force plate horizontally positioned (Bertec FP 4060–15, Bertec Corp., USA) operating at 1000 *Hz* sample rate. From this initial position (90° > *θ*(0) > 85°, measured to the horizontal plane) the rigid body was allowed to drop (Fig 1B) and the vertical and anterior-posterior $\vec{GRF}\left(t\right)$ components were recorded. Six successive trials were conducted to verify the force profile’s repeatability. Data were collected using a 16 bit analogue-to-digital converter (Biopac MP 150, Biopac Systems, Inc., USA) and graphically expressed as function of time.

### Minimum and maximum values of inertia

**Fig 2:**
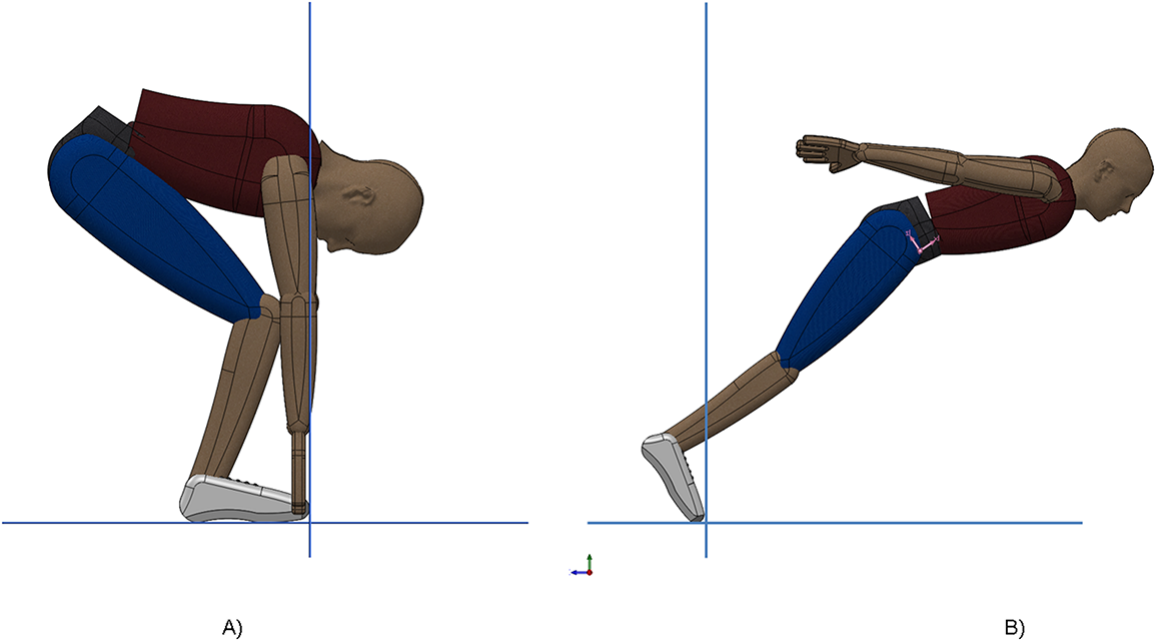
Two rigid articulated body positions mimicking two limit transient body positions: the most contracted (A) and the most extended (B).

Starting with a swimmer model, the minimum and maximum values of the moment of inertia around halluces(*I*_*zz*_), defined by the last component of the inertia tensor matrix, were calculated and are presented below (Table 1). These values were assessed using a model of a rigid articulated body with mass 86.7 *kg*, volume 90.5 *dm* ^3^ and area of 3.28 *m* ^2^ compatible with two transient swimmer’s inter-segmental realistic body positions assumed during the grab start: the most contracted (Fig 2A) and the most extended (Fig 2B) with CM-COP of 0.67 *m* and 1.15 *m*, respectively. The expression “articulated” refers to the reality-based effective transition from the 1^st^ to the 2^nd^ grab start positions. The NASA [13] human body anthropometrical inertial model was used to calculate the *I*_*zz*_ values around halluces in both positions (considering the sagittal symmetry) using SolidWorks (3D CAD, DS Solidworks, Dassault Systèmes S.A., USA).

**Table 1:** Inertia Tensors (*kg m* ^2^) calculated to hallux rotation point in the two rigid articulated body positions.

Rigid articulated body positions	Moment of Inertia matricial components
Most contracted	$\left[\begin{array}{ccc} I_{xx} & I_{xy} & I_{xz}\\ I_{yx} & I_{yy} & I_{yz}\\ I_{zx} & I_{zy} & I_{zz} \end{array}\right]=\left[\begin{array}{ccc} +44.6929 & -14.1389 & +0.0007\\ -14.1389 & +8.1041 & -0.0018\\ +0.0007 & -0.0018 & +50.8178 \end{array}\right]$
Most extended	$\left[\begin{array}{ccc} I_{xx} & I_{xy} & I_{xz}\\ I_{yx} & I_{yy} & I_{yz}\\ I_{zx} & I_{zy} & I_{zz} \end{array}\right]=\left[\begin{array}{ccc} +59.6219 & +54.1270 & -0.0000\\ +54.1270 & +55.9084 & -0.0000\\ -0.0000 & -0.0000 & +113.2425 \end{array}\right]$

### Mathematical model of rigid body fall

The simple rigid body falling mathematical description was conducted using the previously calculated *I*_*zz*_ and the CM locus modelling. Since the motion of the rigid body is a rotation about the contact point on the starting block it is preferable to use polar coordinates.

**Fig 3:**
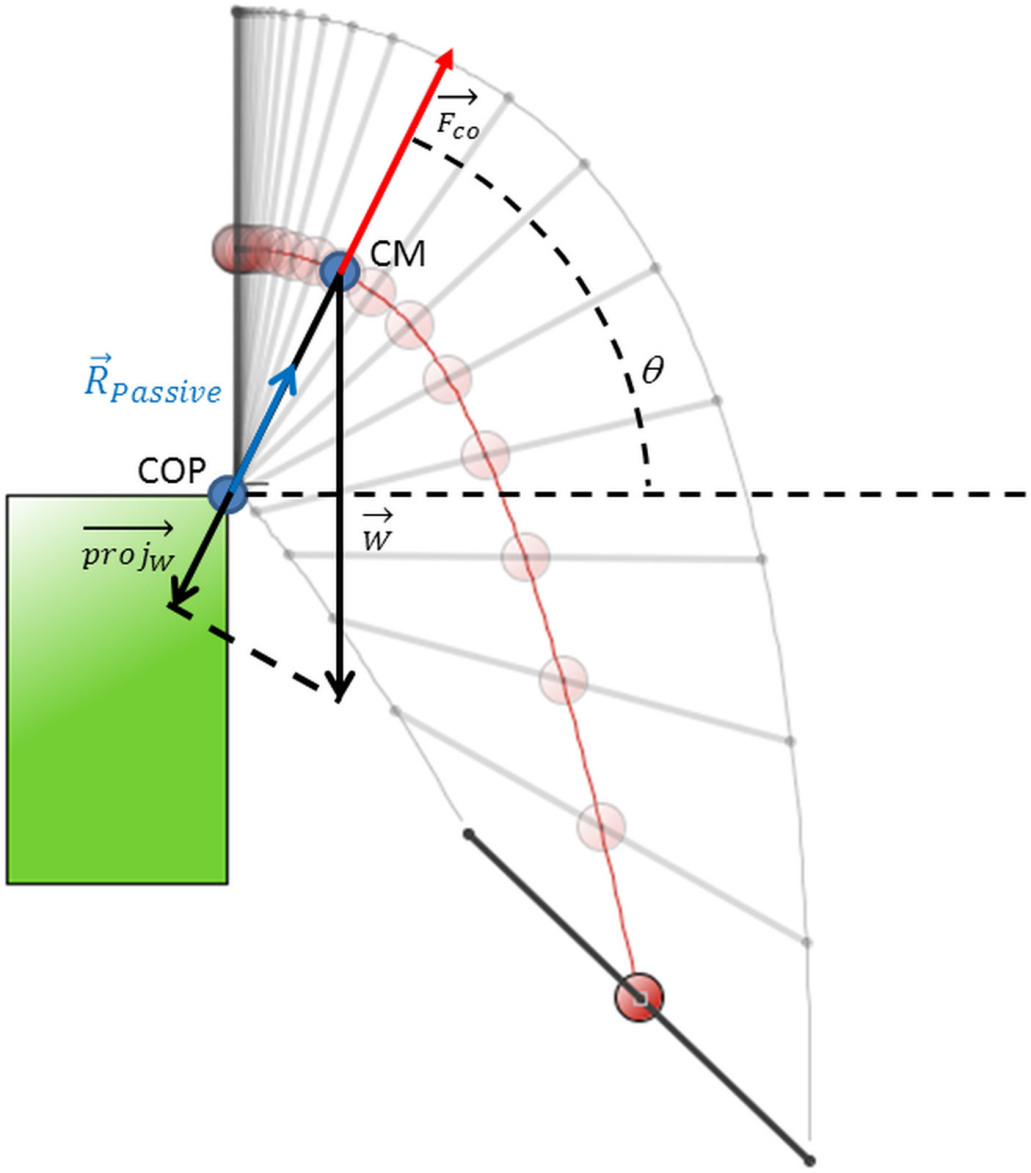
Simulation of the fall of rigid body, representing *θ* the angle to the horizontal, COP the Centre of Pressure, CM the Centre of Mass locus, the $\vec{proj_{\vec{W}}}$ is the weight projection to the CM-COP direction, $\vec{W}$is the rigid body weight, ${\vec{R}}_{Passive}$is the ground reaction force, and $\vec{F_{co}}$ is the centrifugal force.

The forces acting on the radial direction are the projection of the weight $\left(\left\|\vec{{\text{Proj}}_{\vec{W}}}\right\|=m\cdot g\cdot \text{sin}\;\theta \right)$ and the GRF, which in this work is assumed to have only a radial component. Since this force will be called the passive component, to avoid confusion with the measured GRF from the swimmer, it will be termed $R_{Passive}=\left\|{\vec{R}}_{Passive}\right\|$. The vectorial sum of the three forces, the centrifugal force ${\vec{F}}_{CO}$ $\left(\left\|{\vec{F}}_{CO}\right\|=m\cdot \frac{v^{2}}{r_{CM}}=m\cdot r_{CM}\cdot \omega^{2}\right)$, the centripetal force $\left(\left\|\vec{{\text{Proj}}_{\vec{W}}}\right\|\right)$ and *R*_*Passive*_ are in equilibrium along radial position, while in contact, whose effects may also be accounted for by the use of an accelerated referential (Fig 3), that is *R*_*Passive*_−*m* ⋅ *g* ⋅ sin *θ* + *m* ⋅ *r*_*CM*_ ⋅ *ω* ^2^ = 0 or, equivalently, the following statement:

$$R_{Passive}=m\cdot g\cdot \text{sin}\;\theta -m\cdot r_{CM}\cdot \omega^{2}$$ (5)

For the tangential direction, the motion is better described by Newton’s 2^nd^ law in rotation form, that is $\vec{\tau }=\left[I\right]\cdot \vec{\alpha }$, where $\vec{\tau }$ is the sum of the moments of force about the rotational axis, [*I*] is the nine components moment of inertial tensor and $\vec{\alpha }$ is the angular acceleration. In the present case the ground reaction force $\left({\vec{R}}_{Passive}\right)$ produces no moment, as it acts on the rotation axis (COP), and the moment of the weight is due to its tangential component, that is $\left\|\vec{\tau }\right\|=\left\|{\vec{r}}_{CM}\times \vec{W}\right\|=r_{CM}\cdot m\cdot g\cdot \text{cos}\left(\theta \left(t\right)\right)=\left\|\left[I\right]\cdot \vec{\alpha }\right\|$. Halluces axis shares the direction with the angular velocity $\left(\vec{\omega }\right)$ and angular acceleration $\left(\vec{\alpha }=\frac{d\vec{\omega }}{dt}\right)$ vector (i.e. direction *z*, medio-lateral), whose expression in *xyz* reference axis is $\vec{\omega }=\left(\begin{array}{c} 0\\ 0\\ \omega \end{array}\right)$ and $\vec{\alpha }=\left(\begin{array}{c} 0\\ 0\\ \alpha \end{array}\right)$, respectively. In the grab start case, the moment of inertial tensor is practically reduced to *I*_*zz*_, since the modulus of tensor product gives $\left\|\left[I\right]\cdot \vec{\alpha }\right\|=\left\|\left(\begin{array}{c} I_{xz}\\ I_{yz}\\ I_{zz} \end{array}\right)\cdot \alpha \right\|\approx I_{zz}\cdot \alpha$. Since $\left\|\vec{\alpha }\right\|=\frac{d^{2}\theta }{dt^{2}}=\frac{d\omega }{dt}$ the differential equation to be solved is $\frac{d^{2}\left(\theta \right)}{dt^{2}}=-r_{CM}\cdot m\cdot g\cdot \frac{\text{cos}\left(\theta \left(t\right)\right)}{I_{zz}}$. To numerically solve this equation it is converted into two coupled nonlinear differential equations:

$$\left\{ \begin{array}{l} \frac{d\left(\omega \left(t\right)\right)}{dt}=-r_{CM}\cdot m\cdot g\cdot \frac{\text{cos}\left(\theta \left(t\right)\right)}{I_{zz}}\\ \frac{d\left(\theta \left(t\right)\right)}{dt}=\omega \left(t\right) \end{array}\right.$$ (6)

Where *θ*(*t*) and *ω*(*t*) are unknown functions of time, *I*_*zz*_ is the moment of inertia around COP and one can identify the swimmers main anthropometric parameters, namely *m*, *r*_*CM*_ and *I*_*zz*_. One used a Runge-Kutta method (function ode45, The MathWorks Inc, Matlab R2014b) to numerically solve the equations, with initial conditions that were defined as *ω*(0) = 0 *rad* ⋅ *s* ^−1^; $\theta \left(0\right)=\frac{\pi }{2}-0.001$ *rad*, using a modelling software (Modellus 4.01, Modellus, Portugal).

The halluces contact line was considered as the contact locus with the starting block and deformations of the contact areas and tiny COP displacements were discarded.

The two previously obtained rigid articulated body configurations of the inertial tensor and CM position were used to assess the weight torque in the two limiting swimmer configuration (most contracted and most extended) that leads to angular position, angular velocity, angular acceleration (Eq 6). However, contact forces and linear velocity are the observable parameters. It is possible to associate the ${\vec{R}}_{Passive}$ components with *θ*(*t*) and *ω*(*t*). These components are the observable (and therefore, measured) forces while in contact to ground. Eq (7) state force association while Eq (8) state position-velocity $\left({\vec{r}}_{\text{CM}},{\vec{v}}_{\text{CM}}\right)$ respectively, vectorial association.

$$\left\{ \begin{array}{l} R_{\text{v}\_\text{passive}}=m\cdot \left(g\cdot sin\theta -r_{CM}\cdot \omega^{2}\right)\cdot sin\theta \\ R_{\text{h}\_\text{passive}}=m\cdot \left(g\cdot sin\theta -r_{CM}\cdot \omega^{2}\right)\cdot cos\theta \end{array}\right.$$ (7)

$$\left\{ \begin{array}{l} {\vec{r}}_{\text{CM}}=r_{CM}\cdot \left(cos\theta ,sin\theta \right)\\ {\vec{v}}_{\text{CM}}=r_{CM}\cdot \omega \cdot \left(sin\theta ,-cos\theta \right) \end{array}\right.$$ (8)

Eqs (7) and (8) state for the CM kinetics and for the CM kinematics description while in contact to ground with COP as origin of the Cartesian referential frame. The resulting movement should be a pure rotation around the COP.

Knowing that the grab start was selected due to rotations around both halluces axis, any difference of the measured contact force-time curves (incremental or decremental) during the movement compared to the passive force (Eq 7) should be interpreted as the swimmer’s active force effect.

An evidence of this model is that, as it is a pure rotation around COP, the swimmer is only able to perform forces parallel to the CM-COP segment.

### Experimental start protocol

#### Ethics statement

The present study was approved by the Ethics Committee of Faculty of Sport from the University of Porto. All participants provided informed written consent before data collection. The procedures were performed according to the Declaration of Helsinki.

#### Experimental measurements and analyses

Six well-trained swimmers (24.25 ± 3.61 years of age; 1.73 ± 0.08 *m* of height, and 68.19 ± 10.78 *kg* of body mass), were made fully conversant with the protocol. After a standardized warm-up, participants performed three 15 *m* maximal grab start repetitions (3 min resting) over a 3D force plate (Bertec FP 4060–15, Bertec Corp., USA) sampling at 1000 Hz and mounted on a special support designed to replicate a starting block used in international level competitions. A starter device (Omega StartTime IV, Swiss Timing Ltd., Switzerland) was instrumented to simultaneously produce the starting signal and export a trigger signal allowing data synchronization with the acquired $\vec{GRF}\left(t\right)$ curves and analogue-to-digital converted by a 16 bit A/D converter (Biopac MP 150, Biopac Systems, Inc., USA). The block surface angle to the horizontal reference plane (10°) was corrected by applying a suitable rotation matrix and, therefore, vertical vs. horizontal forces were assumed rather than perpendicular vs. anterior-posterior forces.

In order to allow the comparison of the forces produced by swimmers of different masses, the forces (both the passive obtained from the model and the measured *GRF* from the swimmer starting motion) were divided by the respective weight.

**Fig 4:**
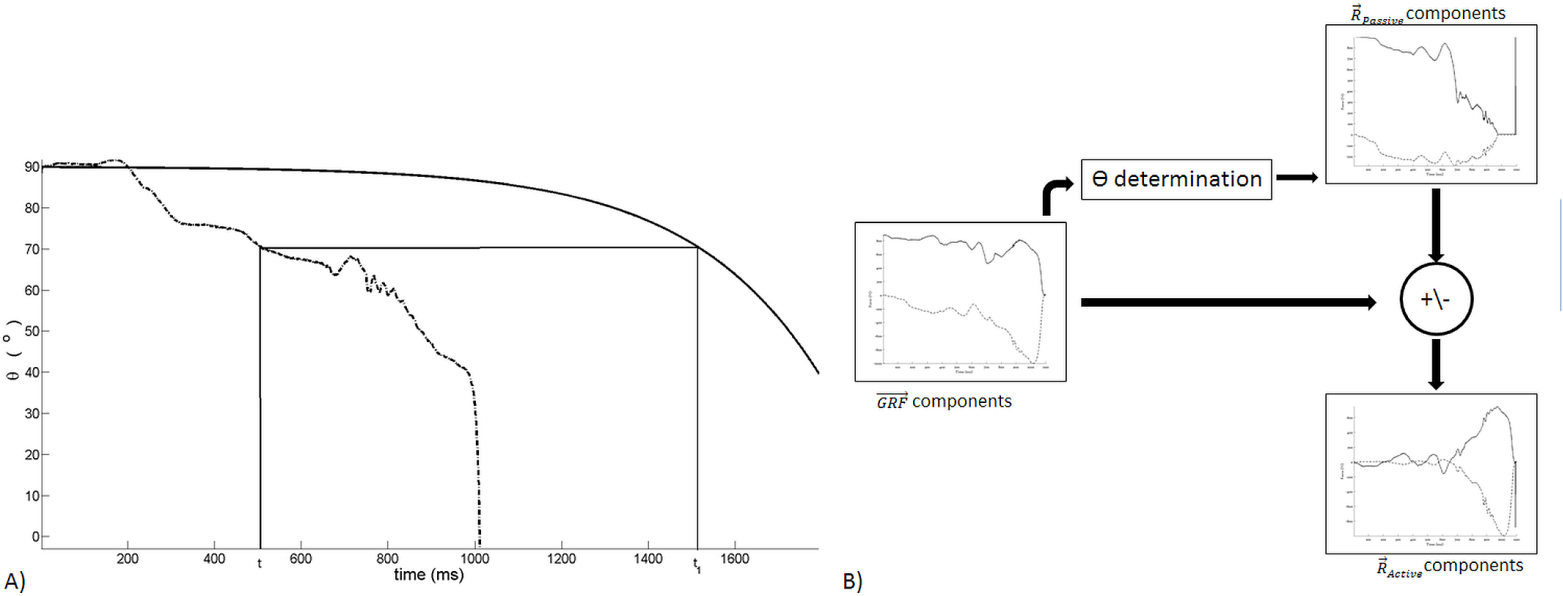
*θ*(*t*_1_) determination to process the $\vec{GRF}\left(\text{t}\right)$ components to split it in ${\vec{R}}_{Passive}$ nd the $\vec{R_{Active}}$ algorithm: graphical description with *θ* generated by the swimmer in dashed-dotted curve and rigid articulated body in continuous curve (panel A) and raw algorithm flowchart (panel B).

Following the experimental protocol the active and passive from raw force splitting tool was applied. The algorithm assumes the perpendicular to CM-COP segment active force unavailability, which means that raw force leads also to a raw *θ* estimator. In first step, we calculate $\theta_{Raw}\left(t\right)=\text{atan}\left(\frac{GRF_{v}\left(t\right)}{GRF_{h}\left(t\right)}\right)$, where *GRF*_*h*_(*t*) and *GRF*_*v*_(*t*) are the horizontal and vertical $\vec{GRF}\left(t\right)$ components, respectively. Simultaneously, it is built the intermediate force-time variables of Eq (9).

$$\left\{ \begin{array}{l} R_{h\_Passive\_i}\left(t_{1}\right)=\frac{m_{swimmer}}{m_{Model}}R_{h\_Passive\_Model}\left(t_{1}\right)\\ R_{v\_Passive\_i}\left(t_{1}\right)=\frac{m_{swimmer}}{m_{Model}}R_{v\_Passive\_Model}\left(t_{1}\right)\\ \theta_{Passive\_i}\left(t_{1}\right)=\text{atan}\left(\frac{R_{v\_Passive\_i}\left(t_{1}\right)}{R_{h\_Passive\_i}\left(t_{1}\right)}\right) \end{array}\right.$$ (9)

where *R*_*h*_*Passive*_*i*_ (*t*_1_) and *R*_*v*_*Passive*_*i*_ (*t*_1_) are the passive reaction horizontal and vertical and *θ*_*Passive*_*i*_ (*t*_1_) is the angle to the vertical. These force values are adjusted, therefore, to the mass of the swimmer but are expressed in the dependency of the unknown time *t*_1_. The *θ*_*Passive*_*i*_ (*t*_1_) angle provides *t*_1_ determination so that minimum of |*θ*_*Passive*_*i*_ (*t*_1_)−*θ*_*Raw*_ (*t*)| at instant *t* is reached. Fig 4A and S1 Files represents the stepwise algorithm for *t*_1_ finding. From *t*_1_ it is built *R*_*h*_*Passive*_ (*t*) = *R*_*h*_*Passive*_*i*_ (*t*_1_) and *R*_*v*_*Passive*_ (*t*) = *R*_*v*_*Passive*_*i*_ (*t*_1_). In the last step, ${\vec{R}}_{Active}\left(t\right)$ is calculated with Eq (10)

$$\left\{ \begin{array}{l} R_{h\_Active}\left(t\right)=GRF_{h}\left(t\right)-R_{h\_Passive}\left(t\right)\\ R_{v\_Active}\left(t\right)=GRF_{v}\left(t\right)-R_{v\_Passive}\left(t\right) \end{array}\right.$$ (10)

Fig 4B diagram depicts the general operations done to split the active S1 Files and passive from the raw force.

### Statistical procedures

Pearson correlation coefficient between experimental rigid body fall and simulated were used in the vertical and horizontal force components. The three force-time curves (raw, active and passive) of each swimmer (i.e., 18 force-time curves for each pair of forces studied) were reported as mean (±*s*) and the variability displayed in each mean curve was assessed by the coefficient of variation.

## Results

### Physical rigid body falling and simulations

**Fig 5:**
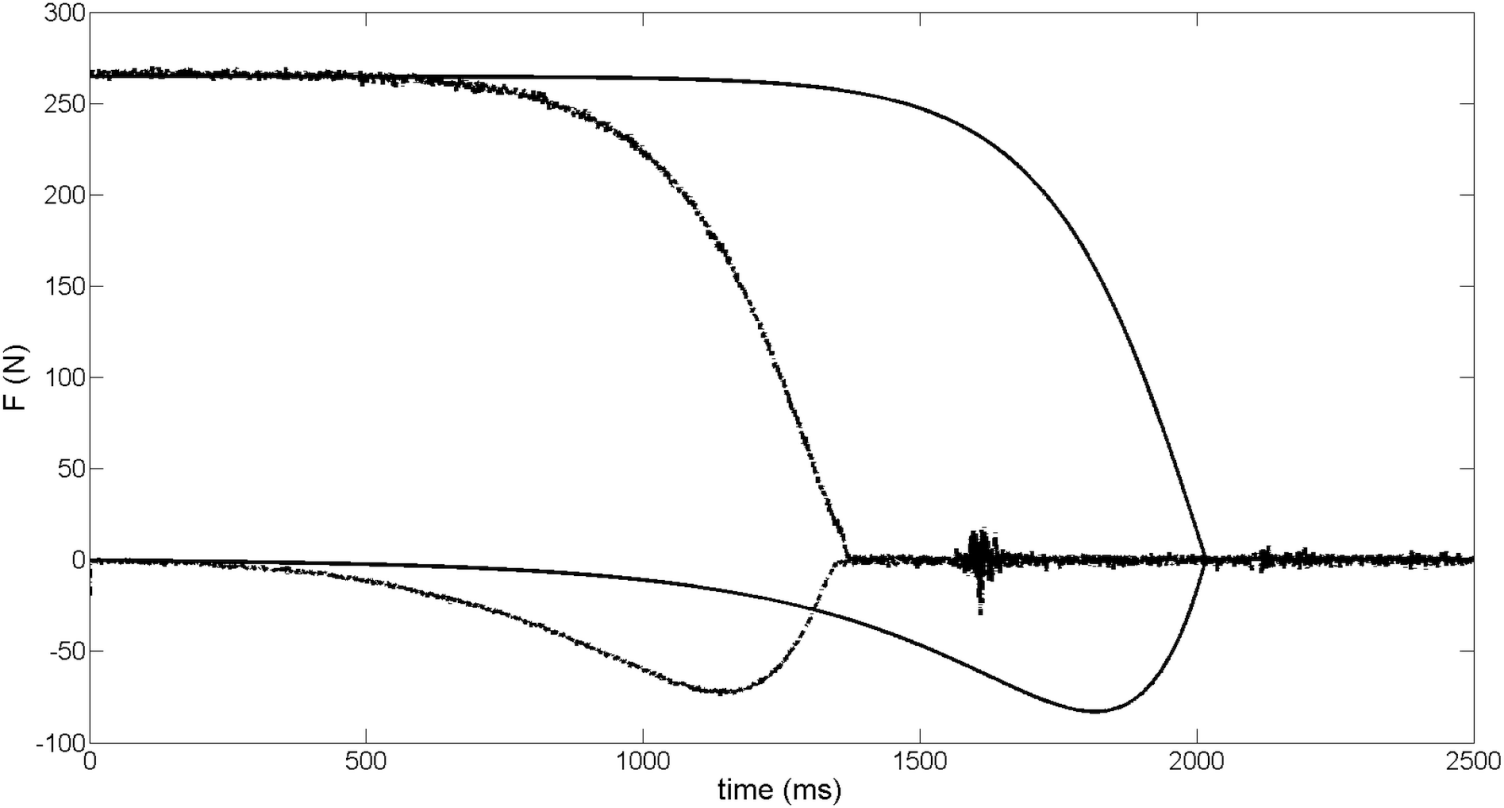
Experimental rigid body falling pattern (discretized lines) and force obtained from the mathematical model of ${\vec{R}}_{Passive}$ (continuous line) pattern. The vertical and horizontal force components, in both cases, are represented. For clarity of representation the horizontal component has been multiplied by -1.

Fig 5 displays the vertical and horizontal components of the $\vec{GRF}\left(t\right)$ measured during the experimental rotating fall of the rigid body, and the respective simulation. For the experimental rotating fall, there was a quasi-stable vertical force-time curve profile up to ~650 *ms* and, subsequently, a monotonic force reduction until the take-off. The horizontal component displayed a stable zero value up to ~150 *ms* and a monotonic increase until ~1150 *ms*, which characterizes a peak of -74 *N* (i.e., ~30% of the body weight considered) before the take-off. Even in the absence of any active propulsion effort, real propulsion can be observed. The force-time curves processed by means of the simulation was similar to the profile observed during the rigid body fall experiment. It was noted a quasi-stable vertical force-time curve profile up to ~1300 *ms* and, subsequently, a monotonic force reduction until the take-off. From the horizontal component, a stable value was displayed up to ~750 *ms* and a monotonic increase until ~1800 *ms* that characterizes a peak of -80 *N* (i.e., 30% of the body weight considered) before the take-off. Correlation for the vertical and horizontal components between experimental and simulated were 0.905 and 0.999 respectively.

The minimum and maximum inertia matrix component *I*_*zz*_ obtained in the respective most contracted and extended rigid articulated body positions (Fig 2) is used to provide correction to the model considered in the rigid body fall simulation. Complete matrix components are presented in Table 1. The *I*_*zz*_ ≫ *I*_*yz*_, *I*_*xz*_ justifies the non-meaningfulness of differences of *I*_*yz*_, *I*_*xz*_ values between both rigid articulated body positions. Inertia *I*_*zz*_ value almost doubles from the most contracted to the most extended rigid articulated body positions.

**Fig 6:**
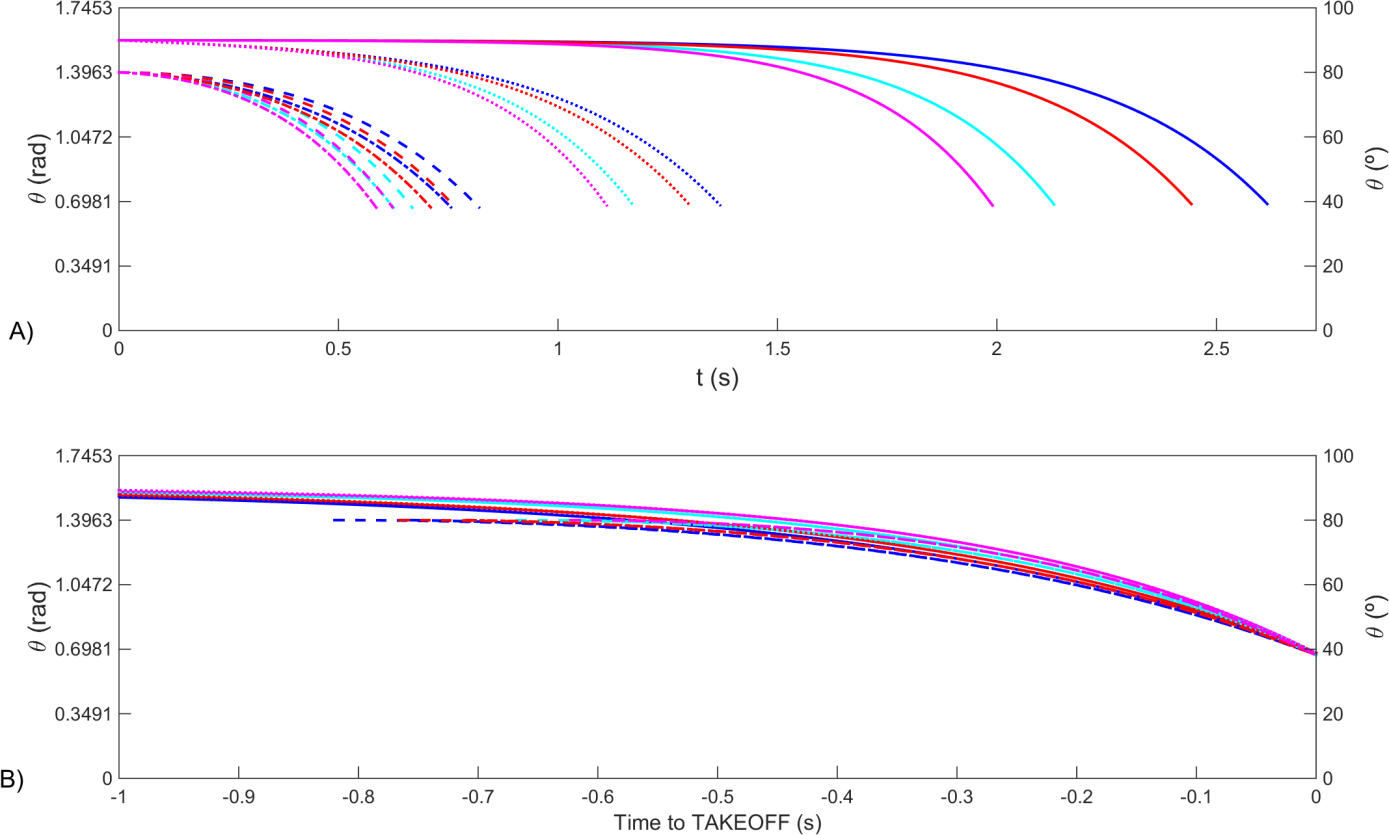
Angle to the horizontal for the mathematical model of rigid body free rotating fall. In both panels the blue and cyan lines for a model with 90 *kg* and the red and magenta for a model with 60 *kg*. The initial conditions are: solid lines: *θ*(0) = 89.9°;ω(0) = 0 *rad* ⋅ *s* ^−1^; dashed lines: *θ*(0) = 80°; ω(0) = 0 *rad* ⋅ *s* ^−1^; dotted lines: *θ*(0) = 90°; ω(0) = -0.1 *rad* ⋅ *s* ^−1^; dash-dotted lines: *θ*(0) = 89.9°; ω(0) = -0.1 *rad* ⋅ *s* ^−1^. In panel A the angles generated have the same initial origin time and in panel B have the same take-off instant.

Fig 6A displays the different simulations for the mathematical model for the angle *θ*, the blue and cyan lines for a model with 90 *kg* and the red and magenta for a model with 60 *kg*. In this panel it is obvious that the time to take-off varies with the inertial properties of the model and the starting conditions. However if we plot in the horizontal axis the time to take-off, then the models all converge to a common area, as depicted in Fig 6B.

**Fig 7:**
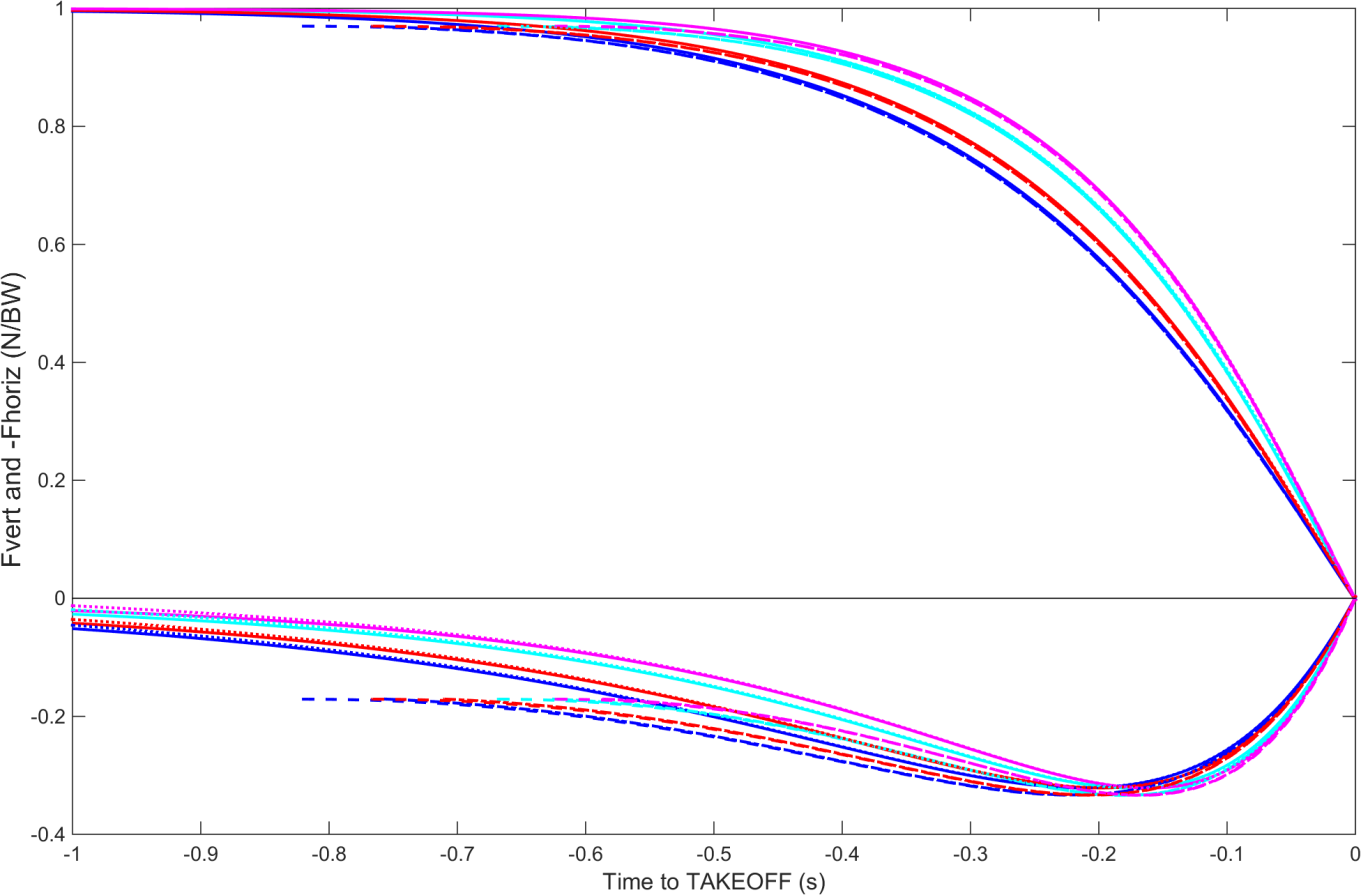
Horizontal and vertical components of the force for the mathematical model of rigid body free rotating fall. In both panels the blue and cyan lines stand for a model with 90 *kg* and the red and magenta for a model with 60 *kg*. The initial conditions are: solid lines: *θ*(0) = 89.9°;ω(0) = 0 *rad* ⋅ *s* ^−1^; dashed lines: *θ*(0) = 80°; ω(0) = 0 *rad* ⋅ *s* ^−1^; dotted lines: *θ*(0) = 90°; ω(0) = -0.1 *rad* ⋅ *s* ^−1^; dash-dotted lines: *θ*(0) = 89.9°; ω(0) = -0.1 *rad* ⋅ *s* ^−1^. For clarity of representation, the horizontal component has been multiplied by -1.

The same is true for the force, both the horizontal and the vertical, as displayed in Fig 7.

### Experimental starting protocol

**Fig 8:**
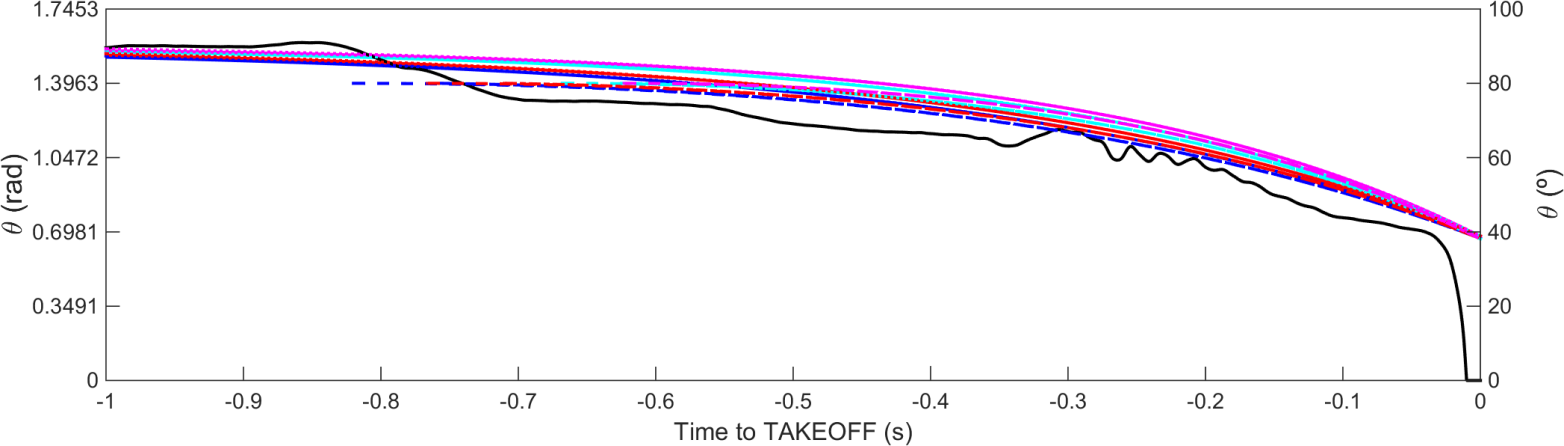
Angle to the horizontal produced by the swimmer, while in contact to block (continuous black line) and angle to the horizontal for the mathematical model of rigid body free rotating fall. The blue and cyan lines stand for a model with 90 *kg* and the red and magenta for a model with 60 *kg*. The initial conditions are: solid lines: *θ*(0) = 89.9°;ω(0) = 0 *rad* ⋅ *s* ^−1^; dashed lines: *θ*(0) = 80°; ω(0) = 0 *rad* ⋅ *s* ^−1^; dotted lines: *θ*(0) = 90°; ω(0) = -0.1 *rad* ⋅ *s* ^−1^; dash-dotted lines: *θ*(0) = 89.9°; ω(0) = -0.1 *rad* ⋅ *s* ^−1^. The angles generated have the same take-off instant.

Fig 8 and S1 Files exhibits the angles (*θ*) generated by the swimmer $\vec{GRF}\left(t\right)$, by the most contracted and by the most extended rigid articulated body falling ${\vec{R}}_{Passive}\left(t\right)$ curves for the equal conditions presented in Figs 6 and 7. The non-smooth swimmer’s *θ* curve exhibits extension values down to ~0°, but only for short time period. The unanimated bodies presented similar *θ* values for take-off and pattern smoothness, and generate a range of values that does not include the swimmer *θ* generated, particularly in the latest values.

**Fig 9:**
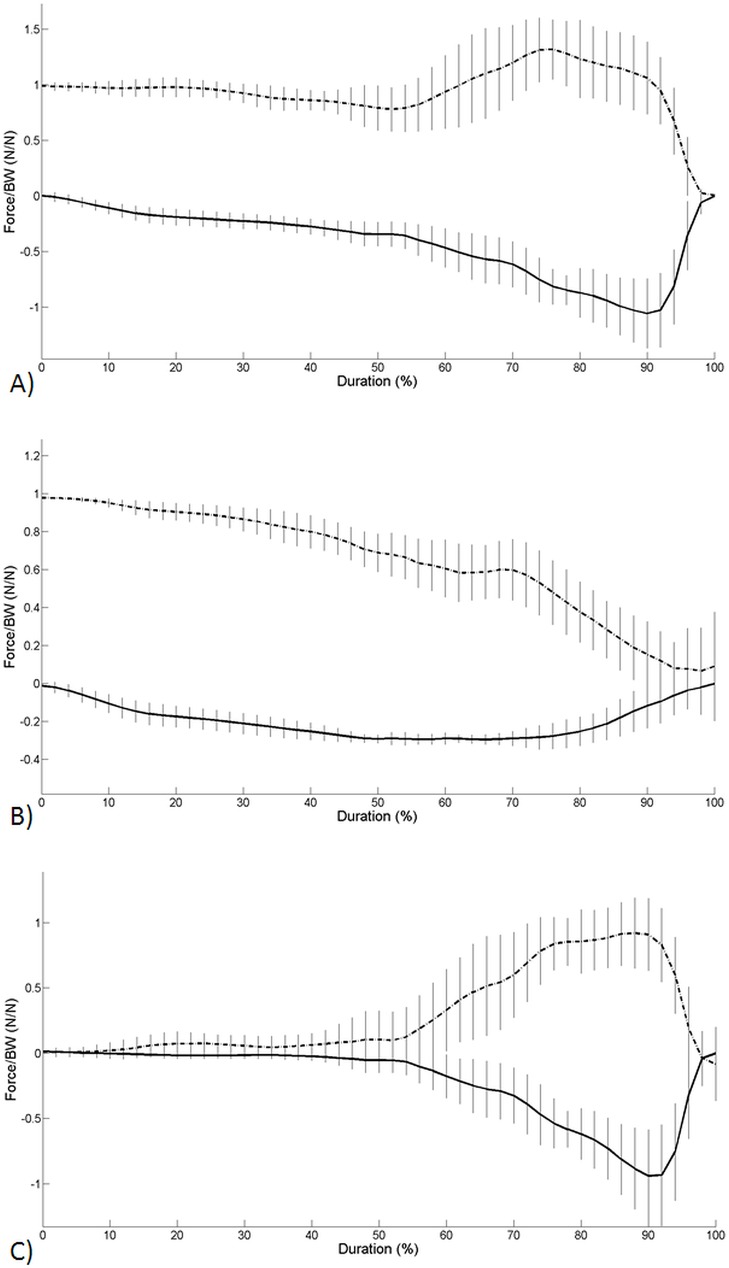
Mean horizontal and vertical (dash-dotted line and continuous line, respectively) force-time curves for the grab start technique, expressed as a percentage of the time period between starting signal and the take-off instant: Raw mean forces (A), passive mean forces (B) and active mean forces (C). The vertical continuous bars denote the local standard variations for each force component. Force data are presented as a fraction of the swimmers’ body weight (BW). For clarity of representation the horizontal component has been multiplied by -1.

The application of the previously mentioned *θ* mapping and determination in each of the 18 individual curves S1 Files lead to the mean raw, passive and active force-time curves and respective (±*sd*) (Fig 9A, 9B and 9C, respectively). Regarding the vertical and horizontal raw force components (Fig 9A), a progressive variability was observed from ~50 to 100% of block time and force values CV of 25.4 and 37.8%, respectively. Concerning both ${\vec{R}}_{Passive}\left(t\right)$ components (Fig 9B), the vertical force showed a progressive variability from ~25 to 100% of block time and force values CV of 16.9%, whereas the horizontal force registered a more restrictive variability (between ~50 to 70% of block time and force values CV of 16.9%). Considering both ${\vec{R}}_{Active}\left(t\right)$ components (Fig 9C), it is verified an abrupt increase in variance from 40% to 100% of block time and force values CV of 67.9% and 66.2%, respectively. An evident symmetry between vertical and horizontal active force mean profiles is noted from the starting signal to the take-off instant (Fig 9C).

## Discussion

In swimming, the start phase is typically divided into the block, flight and underwater sub phases [4,8,12], with the former considered determinant since it initiates the starting action and prepares the following phases [3]. In fact, the study of the force behaviour during the block phase has received considerable attention [4,5,6,7,8,9,10], but researchers have not yet considered the study of dynamometric data based on the physics of the superposition principles, limiting its applicability to regular performance diagnosis [11]. Therefore, we aimed to implement a tool to study the $\vec{GRF}\left(t\right)$ applied on the swimmers during the block phase, splitting ${\vec{R}}_{Active}\left(t\right)$ from ${\vec{R}}_{Passive}\left(t\right)$. The pathways used allowed an appropriate description of both force contributions from the raw data, confirming the hypothesis that swimmer’s forces applied on the starting block are dependent on muscular based biomechanical actions and on the body weight dynamical effects. The application of the splitting algorithm led to a noticeable force variability dependence on ${\vec{R}}_{Active}\left(t\right)$, highlighting that swimmers’ voluntary propulsion is more evident in raw $\vec{GRF}\left(t\right)$ variability than ${\vec{R}}_{Passive}\left(t\right)$.

The current study was conducted with three stepwise determinations with the first two (defined by the force patterns assessment during the falling rigid body) leading to model forces and variable dependencies that were achieved in the following two steps. The force-time curves displayed during the rigid body experiment were similar to the maximum vertical and horizontal force profiles observed during the simulation of the respective phenomena (correlation values, time delays, and maxima/minima values), except the added contact time due to the initial angle (Fig 5). The correlation findings for the vertical force curves are less than 0.95 due to lack of initial data, probably on account of high *θ* accomplishment difficulties. The horizontal peak force observed before the take-off, noticed in the force-time curves of the rigid body experiment and simulation (Fig 5), has a similar profile to that displayed in a previous ventral start study [8]. Swimmers seem to generate the main take-off propulsion between the most contracted and the most extended postures. While mimicking the swimmer postural segment geometries, an unanimated articulated rigid body allows the determination of moment of inertia around COP and limits their respective *I*_*zz*_ values. The changes in inertia moments due to the two different inter-segmental positions (Fig 2) were used in simulations and have shown no effect in the force-time curve profiles allowing the use of *θ* = arctan(*GRF*_*v*_ / *GRF*_*h*_) as a parameter in the CM-COP direction, which was essential for the tool that separated ${\vec{R}}_{Active}\left(t\right)$ from ${\vec{R}}_{Passive}\left(t\right)$ S1 Files (Fig 4). Unanimated *θ* curve exhibited similarity, while the swimmer’s *θ* curve exhibits more irregularities, since the latter depends upon swimmer’s muscular actions. The unanimated curves were similar because *I*_*zz*_ doubles (from 50.8 to 113 *kg* ⋅ *m* ^2^, ratio of 2.22) while *r*_*CM*_ also almost doubles (from 0.67 to 1.15 *m*, ratio of 1.72) making Eq (6) almost invariant. Another supplemental reasoning can generate another quasi-invariance on Eq (6) taking account on the, supposedly, independent anthropometric variables. For instance, consider two ideal swimmers (or any two objects) with different body mass, ascribed *m*_1_ and *m*_2_ but with a similar volumetric mass and with a possible perfect 3D homothety between them. It is possible to perform a transformation between them that applies, as in Eq (11).

$$\left\{ \begin{array}{l} L_{1}\to L_{2}=\sqrt[{3}]{\frac{m_{2}}{m_{1}}}L_{1}\\ V_{1}\to V_{2}=\frac{m_{2}}{m_{1}}V_{1}\\ I_{1}\to I_{2}={\left(\sqrt[{3}]{\frac{m_{2}}{m_{1}}}\right)}^{2}\frac{m_{2}}{m_{1}}I_{1} \end{array}\right.$$ (11)

Where *L* stands for any one-dimensional quantity like *r*_*CM*_ (*m*), *V*(*m* ^3^) for volume and *I*(*kg* ⋅ *m* ^2^) the inertia moment around COP. These transformations, taken simultaneously, leave the Eq (6) with a cos (*θ*) coefficient reduced to 79% if $\frac{m_{2}}{m_{1}}=2$, which is not an usual ratio, while $\frac{m_{2}}{m_{1}}=1.25$ reduces to 93% from the lighter (being the faster) to heavier (being the slower) value. Bigger limb dimensions in the heaviest swimmer could, however, enable contact during time enough to produce more impulse, compensating the loss above mentioned. Eqs (7) and (8) that might differ as *r*_*CM*_ slightly changes, remain seemingly unchanged in function of *θ*. The results of the two mentioned quasi-invariances also motivate us to search what defines the anthropometric difference between swimmers (i.e., intersegment distances and distribution of masses, and specific mass strength or power).

The theta mapping methodology was implemented on a raw swimmer pattern S1 Files (Fig 8) where it is observed the theoretical take-off angle reached before the unanimated curves did. That precise instant should be the matching of the overall curves and posterior force is a pure voluntary force. If the matching of the take-off angles took place then the posterior force exerted should be voluntary force. Also the anterior angle pattern would belong to the neighbourhood of the swimmer’s angle take-off instant the theta earlier values belong to the neighbourhood of the other unanimated curves presented. The theta mapping methodology was also applied on raw swimmers’ force patterns (Fig 9A), allowing the splitting of ${\vec{R}}_{Passive}\left(t\right)$ from ${\vec{R}}_{Active}\left(t\right)$ (Fig 9B and 9C and S1 Files). The raw force-time curves variability was comprised of the respective ${\vec{R}}_{Passive}\left(t\right)$ and ${\vec{R}}_{Active}\left(t\right)$ variability and it was most evidenced in the last 50% of the block time for vertical and horizontal components. This finding was expected since swimmers can effectively propel themselves out of the starting block after the hands leave the handgrips, generating greater resultant impulse with the lower than with the upper limbs [5,6,7,8,10,14]. In fact, ${\vec{R}}_{Active}\left(t\right)$ components also displayed an increased variability from the 40% of the block time, seeming to be the major contributor to the raw variability. The main swimmer’s task has environmental and organism constraints are faced during the most propulsive block instants and subtle differences may distinguish swimmers and swimmers’ trials as a consequence of environmental changes, training procedures or learning phenomena [15]. In contrast, the ${\vec{R}}_{Passive}\left(t\right)$ components are dependent on swimmers’ structure and inertial components, reducing the degrees of freedom involved in swimming start movement and, the consequential variability.

Despite the noticeable contribution of the ${\vec{R}}_{Active}\left(t\right)$ components to the raw force-time curves variability, it should also be considered as their symmetric profile registered from the starting signal to the take-off (Fig 9C), which could indicate a ~45° declination body steering intention. In fact, since the starting signal, swimmers seem to compensate the vertical force reduction as a strategy to falling in a controlled vertical speed, allowing the best angular determination for explosive force during the take-off instants. Force-time curves observed in other swimming start techniques have already displayed qualitatively this symmetry in raw data [8], but no further clarification was exhibited. Several previous raw $\vec{GRF}\left(t\right)$ research findings may lead to a misunderstanding of the real muscular action measurement. The current study evidences the need to consider ${\vec{R}}_{Active}\left(t\right)$ and ${\vec{R}}_{Passive}\left(t\right)$ components to avoid the raw force-time curves masking effects.

Notwithstanding the study’s originality and relevance, some limitations and future research directions should be considered. Firstly, the mathematical model applied still lacks a refined description of part of the rotational angular velocity impact, since the COP might have tiny anterior-posterior movements that were not considered; this indicates the need to include angular positioning and angular velocity mappings for the rigid articulated body passive reaction calculations, particularly knowing that slight angular changes in the beginning can substantially reduce block contact times. We should point out that *ω* cannot vary randomly and has peak values constraints (taking account of its influence in Eqs 7 and 8, which could limit its instantaneous variance, avoiding some of the noticed ringing in ${\vec{R}}_{Passive}\left(t\right)$. This might be an improvement in the development of future algorithms. Secondly, as the lateral responsiveness was not considered, its consistent assessment is recommended to provide detailed dynamometric information for proper forces direction achievement. Calculations could lead to inertial tensor components of mimicking rigid articulated body positions, considering a brand new segment arrangement compatible with the track start, which is also a very commonly used technique. This inertial tensor would lack the proposed grab start sagittal symmetry, and would have, theoretically, dependency on time, but further studies could reveal its minimum and maximum values and how rotation around the hallux would change its dynamical behaviour. The segment arrangement with its origin in the front limb (when rear lower limb takes off) combined with initial angular velocity could lead, once again, to separating assessment, since CM to COP segment and subsequent former grab start considerations could be applied. Future studies should include different intersegmental compatible rigid articulated body transient swimmer’s inter-segmental in ventral and dorsal realistic start body positions to map more *I*_*zz*_ values.

## Conclusions

This is the first study that has implemented a tool to analyse the active and passive vertical and horizontal reaction forces applied by the swimmers during the block phase of a grab start. Experimental events and simulations have confirmed the passive contribution on raw force data and have allowed the separating of the active force component from the swimmers’ force-time curves. The active forces seem to strongly contribute to the raw force variability and denote a vertical and horizontal symmetric profile characteristic of the optimum projection angle to obtain a maximal horizontal displacement range. Future research should consider the active and passive force profiles in different starting techniques for performance advances and aid diagnostics for coaching.

## Supporting Information

S1 Files The six swimmers’ individual force-time curve data. (ZIP)
